# COVID-19, Mental Health, and Chronic Illnesses: A Syndemic Perspective

**DOI:** 10.3390/ijerph20043262

**Published:** 2023-02-13

**Authors:** Kiran Saqib, Afaf Saqib Qureshi, Zahid Ahmad Butt

**Affiliations:** 1 School of Public health Sciences, University of Waterloo, Waterloo, ON N2L 3G1, Canada; 2 Whiting School of Engineering, Johns Hopkins University, Baltimore, MD 21218, USA

**Keywords:** COVID-19, pandemic, mental health, chronic diseases, syndemic

## Abstract

Background: The COVID-19 pandemic is an epidemiological and psychological crisis; what it does to the body is quite well known by now, and more research is underway, but the syndemic impact of COVID-19 and mental health on underlying chronic illnesses among the general population is not completely understood. Methods: We carried out a literature review to identify the potential impact of COVID-19 and related mental health issues on underlying comorbidities that could affect the overall health of the population. Results: Many available studies have highlighted the impact of COVID-19 on mental health only, but how complex their interaction is in patients with comorbidities and COVID-19, the absolute risks, and how they connect with the interrelated risks in the general population, remain unknown. The COVID-19 pandemic can be recognized as a syndemic due to; synergistic interactions among different diseases and other health conditions, increasing overall illness burden, emergence, spread, and interactions between infectious zoonotic diseases leading to new infectious zoonotic diseases; this is together with social and health interactions leading to increased risks in vulnerable populations and exacerbating clustering of multiple diseases. Conclusion: There is a need to develop evidence to support appropriate and effective interventions for the overall improvement of health and psychosocial wellbeing of at-risk populations during this pandemic. The syndemic framework is an important framework that can be used to investigate and examine the potential benefits and impact of codesigning COVID-19/non-communicable diseases (NCDs)/mental health programming services which can tackle these epidemics concurrently.

## 1. Introduction

The term “syndemic” was first conceived by Merrill Singer to describe “synergistically linked” epidemics that coalesce and emerge as a result of adverse social circumstances [1]. A syndemic is defined by the Centers for Disease Control and Prevention (CDC) as “synergistically interacting epidemics” [2] that result in an inordinately increased disease burden due to the clustering of adverse factors; with the interaction between two or more factors more synergistic rather than additive [3]. Syndemic theory emphasizes the socio-structural determinants of health disparities by focusing on critical targets for intervention rather than individuals and connects these socio-structural conditions to synergistic health epidemics [4]. A syndemic is composed of three components, including co-occurring or clustering diseases, adverse interactions between those diseases called biological–biological interaction, and social/environmental and other factors causing or exacerbating the disease. Syndemic is distinguished from other types of epidemics by the presence of both bio–bio and bio–social interactions [4]. In a nutshell, the syndemic approach examines the nature of disease concentrations in populations, as well as interactions, in connection with social, environmental, and economic factors driving this synergism. Furthermore, a syndemic offers predictions about how epidemic interactions add to the illness burden and about how to effectively intervene to reduce this burden [1].

The COVID-19 pandemic can be recognized as a syndemic due to the following: (1) synergistic interactions among different diseases and other health conditions, increasing overall illness burden at every level; (2) emergence and spread of infectious zoonotic diseases and their interactions leading to new infectious zoonotic diseases [5]; (3) social and health interactions leading to increased risks in vulnerable populations and exacerbating clustering of multiple diseases [4]. There is reason to believe that COVID-19 is a syndemic, not only a pandemic, based on the available data: a term used in medical anthropology that combines the concepts of ‘synergy’ and ‘epidemic’, and provides a philosophical backdrop for these disasters. Despite the available reporting on the infrequent fatalities of the young and those without any underlying health condition, the elderly and those with preexisting comorbid health conditions continue to account for the great majority of deaths [6]. People are disproportionately affected by the COVID-19 situation, leading to increased structural disparities not only between social groups and classes, but also between generations [7]. Since there is imposition of new rules, social habits, and mobilizations, it has an impact at both the micro- and macro-system levels [8].

## 2. Review Methods and Search Strategy

A narrative review methodology was chosen to map the body of literature on COVID-19, chronic illnesses, and mental health during the current pandemic, including a greater range of study designs and methodologies, to provide a descriptive overview of the reviewed material. There is no standard protocol for narrative reviews, therefore, a non-systematic electronic literature search was conducted using health-related research databases, including PsycINFO, PubMed, Google Scholar, Web of Science, and Scopus. The search period for relevant studies was conducted in November 2021–March 2022 and the search terms included variations in the terms for the following: ((chronic diseases*) OR (morbidity*) OR (mortality*) OR (deaths*) OR (Severe disease outcome*) AND (severe acute respiratory syndrome coronavirus 2* OR COVID-19* OR SARS-CoV-2* OR COVID-19 pandemic*) AND ((Psychological Stress*) OR (Anxiety*) OR (stress*), OR (Mental Health*) OR (Mental Disorders*) OR (stress disorders*) AND (mental health* OR mental illness*) AND (syndemic*) OR (COVID-19 * AND interaction*) OR (Syndemic* AND coronavirus*) OR (SARS-CoV-2* AND mental health* AND syndemic*)). The database search was conducted on titles, keywords, and abstracts, with “AND” entered to link different categories of search terms. We utilized forward reference searching to identify the references citing these articles and backward reference searching after reviewing the references that were cited in these articles. Non-standardized inclusion and exclusion criteria were used.

Relevant studies were selected only based on the following criteria: (1) focused on the COVID-19 pandemic; (2) they were contextualized to mental health in terms of anxiety and depression only, during the COVID-19 pandemic; (3) they were focused on chronic conditions of interest which included congestive heart failure, hypertension, chronic obstructive pulmonary diseases, asthma, and diabetes only, in terms of either severe disease outcomes or mortality during the pandemic; (4) they were focused on syndemic. All relevant studies published in English were reviewed regardless of their types or designs, and all countries of origin were eligible for inclusion. Articles were excluded if they were (1) published outside the search period, (2) did not focus on anxiety and depression during COVID-19 and reported other mental health conditions, (3) focused on other chronic conditions, (4) were irrelevant to either the COVID-19 pandemic impact on mental health, chronic conditions, and syndemic. Finally, the included articles (*n* = 75) were peer-reviewed research articles, perspectives, reviews, editorials, and commentaries. These articles were reviewed, and relevant data were extracted to report the key findings in the following sections.

## 3. COVID-19 and Mental Health

COVID-19 and the mental health crisis caused by it, references [9,10] are two concurrently occurring epidemics emerging together in real-time, with possible linkages with one another. Due to multiple factors, including uncertainty, loneliness, financial worries, disruption of supply chains, and a lack of access to health care, the COVID-19 pandemic has evolved into a syndemic. Whether it is the direct or indirect psychological and social impacts of the COVID-19 pandemic, it is obvious that it could have a long-term impact on the general population health. While the virus might be potentially deadly, it is a threat to livelihood and social connections that is creating a generational mental health crisis [11,12].

The COVID-19 pandemic is having an extreme impact on all aspects of society. It is believed that stress is a common factor impacting the syndemic of disease and health by influencing social, environmental, and political determinants [4]. There is an increased incidence of fear, insecurity, anxiety disorders, depression, and suicide during these historical periods of stress [13]. Fear-induced overreaction was reported among the general population during and after similar epidemics of SARS in 2003 and Ebola in 2014 [14,15]. Not only this, several psychiatric disorders like anxiety, depression, and post-traumatic stress disorder were reported among SARS and Ebola epidemic survivors and healthcare workers [16,17,18]. Changes in our daily life routines, job loss, loneliness, financial struggle, and grieving over the death of loved ones are all likely to negatively impact many people’s mental health and wellbeing now and in the future.

The COVID-19 pandemic is having an acute and long-term negative impact on anxiety, depression, psychosis, and suicidal ideation, among other clinical and drug use disorders [19]. It is evident that the emotional and mental health impact of COVID-19 and quarantine is much, much more than the fear of contracting the virus [20]. A study reported that 34.1% of participants with an experience of quarantine reported psychological symptoms like stress, anxiety, insomnia, and depression as compared to those who did not experience quarantine (27.3%), indicating the adverse impact of COVID-19 and quarantine measures on mental health [21]. Many of the expected outcomes of quarantine, as well as the associated social and physical separation measures, are important risk factors for mental health problems [22]. A few of these examples include self-harm, suicide addiction, substance abuse, domestic and child abuse, and psychosocial risks due to loneliness, financial stress, and unemployment [23,24]. Uncertainty, boredom, loneliness, loss of freedom, and separation from loved ones, can create dramatic effects [25,26]. During these extraordinary circumstances, it is expected to witness anxiety and coping responses to stress, but at the same time, it is suspected that prevalence of anxiety, depression, and the number of people engaging in harmful behaviors will rise in the future.

The public health measures implemented to limit the spread of COVID-19 have affected all segments of the population. Globally, surveys documenting COVID-19 mental health repercussions have specified certain groups who may be more vulnerable to the pandemic’s psychological consequences. Women, younger age cohorts, those with economic instability or lower income, those who have lost income owing to the pandemic, those who live with others, and those who perceive a higher risk of COVID-19 infection are among the at-risk groups [27,28,29]. Because of the scope of the COVID-19 pandemic, it may be difficult to extrapolate results from previous studies to the present. Prior infectious disease outbreak research has tended to focus on specific populations (e.g., affected patients, health care workers, and persons who have been quarantined), with fewer studies focusing on broader general-population samples [30,31,32]. Despite the fact that the effects of COVID-19 on mental health have not yet been studied systematically, it is expected to have an adverse impact on mental health necessitating immediate action [33,34].

## 4. COVID-19 and Chronic Diseases

COVID-19 impacts almost every system of the human body, including the respiratory, neurological, cardiovascular, renal, gastrointestinal, musculoskeletal, and hematological systems [35]. People with existing health conditions, the elderly, and those with weak immune systems, face a considerable risk from COVID-19 [36]. Co-occurrence of physical health conditions and mental conditions may muddle diagnosis, treatment, and disease progression. For example, heart failure as well as chronic obstructive pulmonary disorder (COPD) may either disguise or reflect symptoms of anxiety, depression, and post-traumatic stress disorder, making their diagnosis improbable [37]. Furthermore, there is an increased risk of mental health issues, stress, and functional impairment due to underlying physical health conditions [38]. Likewise, the higher rates of chronic conditions, including diabetes and hypertension have been reported in those suffering from severe mental disorder [39]. Several research studies have indicated a direct link between chronic diseases with mental health issues like depression [40,41] in the general population [42,43].

It is acknowledged that the interaction between physical and mental health is convoluted, which can worsen the course of both illnesses [44]. Similar interactions have been identified in acute and severe cases of COVID-19, diabetes, and depression. It appears that diabetes promotes viral entry into human cells through certain pathways, while COVID-19 worsens diabetes by stimulating an aggressive damaging immune response [45]. On one hand, co-existence of diabetes and depression cause adverse effects on both morbidity and mortality [46]; while on the other hand, there is a 1.5-fold increase in mortality in people with diabetes due to coexisting depression [47].

COVID-19 also poses a threat to those suffering from cardiopulmonary disease [48] causing hypoxemia in approximately 15–20% of the patients requiring ventilator support in critical conditions [49]. Though COVID-19 infections are not directly linked to asthma, patients with moderate-to-severe or uncontrolled asthma are more likely to be hospitalized from COVID-19 [50], indicating that asthma may be an important risk factor for COVID-19 severity and hospitalization. Smokers who are asthmatic and elderly, in particular, are at an increased risk of severe disease if infected with COVID-19 [51]. In individuals suffering from obesity and hypertension, the immune responses are activated and aggravated by chronic stress and biological aging processes [52,53,54]. A high case fatality is connected to patients suffering from COVID-19 and uncontrolled high blood pressure [55]. A Chinese study reported a 6% case fatality rate (CFR) in 23% of hypertensive COVID-19 cases, and the number continued to increase due to pandemic anxiety [56]. There is a risk of developing acute coronary syndrome in cardiovascular disease (CVD) in patients with COVID-19. It is still not clear about the direct relationship and the mechanism of interaction between COVID-19 and CVD as a compromised immune system was observed in patients with CVD suffering from COVID-19 [57].

With regards to renal disease, due to an increase in ACE-2 expression, COVID-19 disease is severe in patients with renal diseases [58]. The weak immune response is the main risk factor for developing COVID-19 infection in those with malignancies. Factors responsible for enhanced liver damage in COVID-19 patients include drug toxicity, stress, and systematic inflammatory response, paving the pathway for severe hepatic diseases [59]. The impact of COVID-19 on the nervous system and the causes of neurological symptoms experienced by COVID-19 patients are still unknown. According to research, headaches are the most common initial neurological symptom and one of the most prevalent in people diagnosed with COVID-19 infections while the incidence of headaches varies and falls within a wide range of 10–70% [60,61,62,63].

Most non-communicable diseases (NCDs) including diabetes, cardiovascular disease, hypertension, asthma, have been reported as risk factors for severe COVID-19 disease causing an increase in mortality rate and death in most COVID-19 patients and is attributed to the pre-existing comorbidities [55]. Chronic disease patients have been impacted by the COVID-19 pandemic both directly and indirectly. Along with morbidity and mortality, high rates of community spread and numerous mitigation measures, such as the advice to stay at home, have disturbed lives and led to suffering in social and economic spheres [64]. In addition, the ability to prevent or control chronic diseases has been hampered by this pandemic, which has also raised concerns about the security of healthcare access [65].

## 5. COVID-19, Mental Health, and Chronic Diseases

According to the World Health Organization (WHO), 71% of all deaths per year are attributed to a syndemic associated with mental health conditions and NCDs [66]. There is a connection between mental health challenges, the COVID-19 pandemic related morbidity, and mortality caused by the disease itself as well as being related to mitigation activities such as stay-at-home orders and social distancing [67]. Despite the fact that there is not much known about the novel coronavirus, there is evidence of multi-organ-system involvement and syndemic interactions with various NCDs in patients suffering from COVID-19 [68,69,70]. Several risk factors have been identified during the COVID-19 pandemic including pre-existing health conditions as well as adverse social determinants of health that leave many susceptible to the disease [71,72].

The synergism between COVID-19 and NCDs is of reciprocal nature [73]. On the one hand, pre-existing NCDs, including diabetes mellitus, cancer, cardiovascular disease, and chronic obstructive pulmonary disease are found to be associated with poor prognosis, higher risk of complications, increase in intensive care unit admissions, and thereby increased mortality among patients with COVID-19 [74]. While on the other hand, those who survive COVID-19 are more prone to develop or have further long-term chronic and mental health conditions [75]. The elevated rates of NCDs and COVID-19 suggest an inclusive syndemic health burden, pointing towards the intersection of both with social determinants of health. There is considerable evidence hinting towards NCDs as a major factor responsible for adverse outcomes as a result of COVID-19 infection [75,76] and vice-versa, while COVID-19 also contributes to the neglect of NCDs [75].

Managing and treating COVID-19 patients is still a major challenge for health care staff as effective antiviral medications are not yet available. This is because although select antivirals have proved efficacy in improving clinical outcomes in COVID-19 patients, there are mixed results for their efficacy in reducing mortality [77]. COVID-19 in individuals with comorbidities leads to a nexus of increased morbidity and higher mortality. It causes a detrimental impact on lungs, heart, and kidneys in individuals suffering from conditions like diabetes, CVDs, and COPD. All these complications have a deleterious impact on the patient’s health due to acute respiratory distress syndrome, heart failure, renal failure, multiple organ failure, shock, and ultimately mortality [78,79]. Despite the fact that most COVID-19 patients develop mild disease, 20% of patients require hospitalization, with 5–8% developing severe symptoms and necessitating intensive care and intensive care unit (ICU) admission [80]. There is limited accurate data about exact duration of ventilation but there is an increased need of constant mechanical ventilation for two weeks or more, to enable patients to breathe normally [55]. Factors influencing ICU admissions include predisposing factors like age and comorbidities. Data from different countries show that highest rates of ICU admissions were recorded in the USA at 81%, Italy 5–12%, and 7–26% in China [81,82,83].

More than half a million people have died because of the COVID-19 epidemic, which has caused severe personal and societal losses [84]. In addition to the physical cost, the emotional consequence is still not entirely clear. The effects have been especially significant for people with chronic illnesses [69,85]. Conditions that raise the risk for COVID-19-related severe illness include heart disease, diabetes, cancer, chronic obstructive lung disease, chronic renal disease, and obesity [86]. Finally, since February 1, 2020, more people have died from dementia, cardiovascular, and other causes in addition to COVID-19-related mortality. This increase may be attributable to COVID-19 related deaths or to the virus’s unintended consequences, such as underutilization of or burden on the healthcare system [87].

An estimated 14.9 million excess deaths directly associated with COVID-19 have been reported by the WHO between January 2020 and December 2021 [88]. This calls for attention to explore excess deaths beyond those directly due to COVID-19, in assessment of the full impact of the COVID-19 pandemic on overall mortality [89]. Along with the financial crisis due to the COVID-19 pandemic, there are also indications of mortality due to “deaths of despair”. The diseases of despair are three classes of behavior-related medical conditions that include drug overdose, suicide, and alcoholic liver disease. These deaths are associated with a poor quality of life, including deterioration in emotional and physical health, resource constraints, and severe mental illness. Alcohol is a significant factor in mortality brought on by despair and is thought to be responsible for 15% of all drug overdoses, 26% of suicides, and 50% of liver disease deaths [90]. The pandemic mitigation strategies have also reduced social interaction due to physical distancing. Since human social connection is a strong reinforcer, a lack of it can lead to loneliness, dysphoria, despair, and general malaise. Negative emotional states have been exacerbated for many because of the ongoing COVID-19 pandemic, which has also caused social isolation, the loss of loved ones, loss of livelihood, and drinking to cope. It is possible that rising pandemic-related stress and increased drug use may fuel an increase in mortality from overdoses, suicide, and alcohol-related liver disease since the pandemic started against the backdrop of rising deaths from despair in the United States [91]. These fatalities result from an increase of behavioral medical conditions in people suffering from despair due to a dreary outlook of their long-term socio-economic prospects [92]. It has been suggested that this may affect ‘at-risk’ groups as well as those facing barriers to access care, having both short- and long-term implications for mental health and substance use. This is raising concerns over both cases and additional deaths, calling for action for those already-vulnerable populations at greater risk of contracting COVID-19 [93]. Certain populations, including specific race and ethnic groups such as African Americans, Hispanics, and Native Americans, as well as those with poor socioeconomic status, are suffering from chronic diseases, COVID-19 disease, hospitalization, and mortality [94]. Due to exposure to inadequate social determinants of health (SDoH), these populations are more at risk. Where people live, work, and play has an impact on their health, and SDoH can lead to barriers that exacerbate inequalities. Numerous factors including institutional racism, lack of access to healthy food, lack of safe and cheap housing, lack of education, kind of employment, inadequate or no health care, and other factors all have an impact on a variety of health outcomes [95,96,97]. The COVID-19 pandemic has exposed fundamental causes of health disparities and aggravated them. The current situation necessitates a holistic approach and effective coordination of health services during such health emergencies [98].

## 6. Syndemic Approach—A Way Forward

The concept of syndemic is not new; it has been applied to understand various issues, including health disparities, the dynamics of migrant health, malnutrition, violence, substance abuse, mental health, sexual health, infectious diseases like HIV/AIDS, tuberculosis, and non-communicable diseases (such as diabetes mellitus, cardiac and pulmonary), over the years [46,99,100,101]. The syndemic approach has gained popularity and has been utilized to study both global health and global mental health over the past 20 years [102]. Initially the syndemic approach was focused on vulnerable communities in only high-income countries (HICs) [103], but lately, the syndemic approach has been applied to low- and middle-income countries (LMICs) too [104,105,106]. Researchers, such as Mendenhall et al. [46]. and Irene et al. [107] have studied mental health conditions along with NCDs in those countries where infectious diseases or disease-specific interventions are a priority for research and policy [104].

Syndemic theory argues that the pandemic might exacerbate disparities in the social, political, and economic conditions that elevate the risk of disease concentration in vulnerable populations. It considers the synergistic effects of social, environmental, and political factors affecting etiology and prognosis, at both population and individual level, moving beyond comorbidity [108] and helps us understand how these factors contribute and influence the onset and clustering of diseases [109,110]. In addition to disease concentration, disease interaction is a dominant feature of a syndemic. These interactions are thought to worsen pandemic consequences by having multiplicative rather than additive impacts. The syndemic interactions play a prominent role in defining the trajectories and intensification of infectious diseases [3,111]. The significance of syndemic thinking is emphasized by the existence of synergistic effects in the short and long term [112].

**Figure 1:**
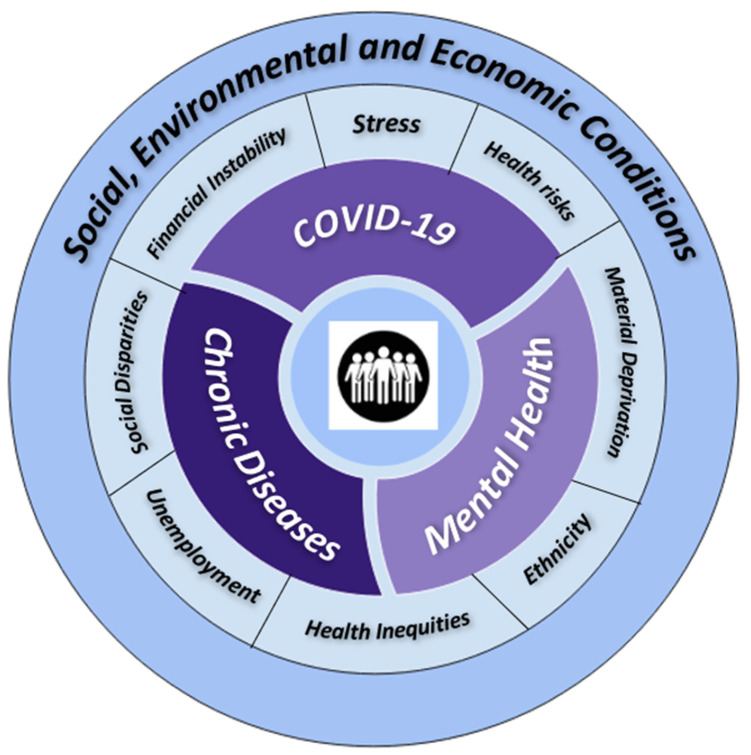
Conceptual framework for COVID-19, Mental Health, and Chronic diseases Syndemic.

The COVID-19 outbreak continues to have an impact on populations, especially those living in poverty, the elderly, people with disabilities, children, and indigenous peoples. It is most harmful to those individuals who belong to the social groups that are most at risk. Early pandemic data suggest that the poor are disproportionately paying the costs of the virus’s effects on their health and economy. For instance, homeless individuals are more vulnerable in addition to refugees and migrants, who suffer disproportionately from the epidemic and its aftermath due to restrictions on movement and a lack of work prospects. COVID-19 teaches us that social and biological risk are closely intertwined. Viruses may infect individuals, but pandemics impact entire populations. Like previous pandemics, this disease is a reflection of political, economic, and social circumstances, and the concept of syndemic helps us grasp these interactions [113]. The challenging social, economic, and power disparities, inequities in the distribution of health risks and resources, result in disease concentration in a specific vulnerable population, while the biological interactions point towards COVID-19 being a syndemic (Figure 1). The aggregation of disease on a background of social and economic disparity exacerbates the adverse effects of already existing comorbidities [114]. People infected with COVID-19 have suffered severe outcomes due to pre-existing diseases including hypertension, diabetes, and respiratory problems., while the virus spread has been fueled by the existing social and health inequalities, and a fragmented health-care system. Synergistic failures have resulted in more death and devastation than in many other situations [109] since it has been reported that COVID-19 has killed more people than severe acute respiratory syndrome (SARS) and Middle East respiratory syndrome (MERS) combined [115].

The COVID-19 pandemic has evolved into an extreme kind of syndemic, a pan-syndemic [116], which has exposed inequities by increased disease burden and death compressed within a short time span globally [71]. Higher mortality rates have been reported in underserved populations throughout the world in both developed and developing countries [117,118,119]. The worldwide excess deaths in 2020 have surpassed the already shocking number of deaths directly attributed to COVID-19 but syndemic interactions are likely to be blamed [120]. Pre-existing patterns of uneven distribution of NCDs reflect well-known societal trends, especially along social class, and ethnic lines. COVID-19 status has changed from pandemic to syndemic within this nexus [121]. An increase in the incidence and prevalence of anxiety and depression is as a result of the COVID-19 pandemic, and experts are already warning of an “echo pandemic” [122]; a new term that describes the possibility of widespread mental health issues that resemble COVID-19 in scale [123].

Since the coronavirus pandemic is an epidemiological and psychological crisis, there is some understanding of its effect on populations, and more research is underway, but the syndemic impact of COVID-19 and mental health on underlying chronic illnesses among the general population is not completely understood. Originally syndemic theory was comprised of disease clustering, interaction between diseases, and the wide range of social factors giving rise to them, but it is a complex, multilevel phenomenon (Figure.1). The significance of this approach lies in its ability to predict how the interplay among different epidemics can magnify overall disease burden, which differs if each disease would have been examined individually. The collective factors between multiple epidemics can be interpreted with a syndemic approach. There is a gap to explore and investigate how epidemics interact both at the level of populations and individuals during this COVID-19 pandemic. The application of syndemic theory in the COVID-19 pandemic may guide public health planners to design effective interventions to mitigate disease burden [108]. There is a need to generate evidence to support appropriate and effective interventions to improve the health and psychosocial wellbeing of vulnerable populations during this pandemic [108]. In order to protect the health of our communities, the syndemic and complex nature of the current pandemic calls for a unique and sophisticated approach.

## 7. Conclusions

The syndemic framework is an important and robust model with the ability to investigate and examine the potential benefits and impact of codesigning COVID-19/NCDs/mental health programming services, which can tackle these pandemics concurrently. A syndemic perspective to COVID-19 can play a pivotal role in helping health policy makers and public health program implementers in their endeavors to improve the overall health of populations.

## Data Availability

Not applicable.

## Abbreviations

Centers for Disease Control and Prevention (CDC), coronavirus disease 2019 (COVID-19), chronic obstructive pulmonary disease (COPD), cardiovascular disease (CVD), non-communicable diseases (NCDs).

## References

[1] Tsai A.C. (2018). Syndemics: A theory in search of data or data in search of a theory? Social Science and Medicine. Soc. Sci. Med..

[2] Definitions|PCSI|NCHHSTP|CDC (2014). National Center for HIV/AIDS, Viral Hepatitis, STD, and TB Prevention. https://www.cdc.gov/nchhstp/programintegration/definitions.htm.

[3] Singer M., Bulled N., Ostrach B., Mendenhall E. (2017). Syndemics and the biosocial conception of health. Lancet Lancet.

[4] Singer M., Clair S. (2003). Syndemics and Public Health: Reconceptualizing Disease in Bio-Social Context. Med. Anthr. Q..

[5] Lawler O.K., Allan H.L., Baxter P.W., Castagnino R., Tor M.C., Dann L.E., Hungerford J., Karmacharya D., Lloyd T.J., López-Jara M.J. (2021). The COVID-19 Pandemic Is Intricately Linked to Biodiversity Loss and Ecosystem Health. Lancet Planet. Health.

[6] WHO-China Joint Mission (2020). Report of the WHO-China Joint Mission on Coronavirus Disease 2019 (COVID-19). Braz. J. Implantol. Health Sci..

[7] Dosi G., Soete L. (2021). On the syndemic nature of crises: A Freeman perspective. Res. Policy.

[8] Duarte M de Q., Santo MA da S., Lima C.P., Giordani J.P., Trentini C.M. (2020). COVID-19 and the impacts on mental health: A sample from Rio Grande do Sul, Brazil. Cienc e Saude Coletiva.

[9] Pies R.W. (2020). Is the Country Experiencing a Mental Health Pandemic?. Psychiatr. Times.

[10] Centre for Addiction and Mental Health (2021). The Mental Health Crisis Is Real|CAMH. Centre for Addiction and Mental Health. https://www.camh.ca/en/driving-change/the-crisis-is-real.

[11] Mertens G., Gerritsen L., Duijndam S., Salemink E., Engelhard I.M. (2020). Fear of the coronavirus (COVID-19): Predictors in an online study conducted in March 2020. J. Anxiety Disord..

[12] Su Z., McDonnell D., Wen J., Kozak M., Abbas J., Šegalo S., Li X., Ahmad J., Cheshmehzangi A., Cai Y. (2021). Mental health consequences of COVID-19 media coverage: The need for effective crisis communication practices. Glob. Health.

[13] Cândido E.L., Júnior J.G. (2021). COVID-19 Syndemic, Government, and Impact on Mental Health: A Brazilian Reality. Front. Psychiatry.

[14] Person B., Sy F., Holton K., Govert B., Liang A., Team S.C.O., Garza B., Gould D., Hickson M., McDonald M. (2004). Fear and Stigma: The Epidemic within the SARS Outbreak. Emerging Infectious Diseases. Cent. Dis. Control Prev. (CDC).

[15] Shultz J.M., Cooper J.L., Baingana F., Oquendo M.A., Espinel Z., Althouse B.M., Marcelin L.H., Towers S., Espinola M., McCoy C.B. (2016). The Role of Fear-Related Behaviors in the 2013–2016 West Africa Ebola Virus Disease Outbreak. Curr. Psychiatry Rep..

[16] Blakey S.M., Kirby A.C., McClure K.E., Elbogen E.B., Beckham J.C., Watkins L.L., Clapp J.D. (2019). Posttraumatic Safety Behaviors: Characteristics and Associations with Symptom Severity in Two Samples. Traumatology.

[17] Gardner P.J., Moallef P. (2015). Psychological impact on SARS survivors: Critical review of the english language literature. Canadian Psychology. Can. Psychol. Assoc..

[18] Mak I.W.C., Chu C.M., Pan P.C., Yiu M.G.C., Chan V.L. (2009). Long-term psychiatric morbidities among SARS survivors. Gen. Hosp. Psychiatry.

[19] Wildwing T., Holt N. (2021). The neurological symptoms of COVID-19: A systematic overview of systematic reviews, comparison with other neurological conditions and implications for healthcare services. Ther. Adv. Chronic Dis..

[20] Barbisch D., Koenig K.L., Shih F.-Y. (2015). Is There a Case for Quarantine? Perspectives from SARS to Ebola. Disaster Med. Public Health Prep..

[21] Wang Y., Shi L., Que J., Lu Q., Liu L., Lu Z., Xu Y., Liu J., Sun Y., Meng S. (2021). The impact of quarantine on mental health status among general population in China during the COVID-19 pandemic. Mol. Psychiatry.

[22] Brooks S.K., Webster R.K., Smith L.E., Woodland L., Wessely S., Greenberg N., Rubin G.J. (2020). The psychological impact of quarantine and how to reduce it: Rapid review of the evidence. Lancet.

[23] O’Connor R.C., Nock M.K. (2014). The psychology of suicidal behaviour. Lancet Psychiatry.

[24] John A., Glendenning A.C., Marchant A., Montgomery P., Stewart A., Wood S., Lloyd K., Hawton K. (2018). Self-harm, suicidal behaviours, and cyberbullying in children and young people: Systematic review. J. Med. Internet Res..

[25] Li S.W., Wang Y., Yang Y.Y., Lei X.M., Yang Y.F. (2020). Analysis of influencing factors of anxiety and emotional disorders in children and adolescents during home isolation during the epidemic of novel coronavirus pneumonia. Chin. J. Child. Health.

[26] Cao W., Fang Z., Hou G., Han M., Xu X., Dong J., Zheng J. (2020). The psychological impact of the COVID-19 epidemic on college students in China. Psychiatry Res..

[27] Hyland P., Shevlin M., McBride O., Murphy J., Karatzias T., Bentall R.P., Martinez A., Vallières F. (2020). Anxiety and depression in the Republic of Ireland during the COVID-19 pandemic. Acta Psychiatr. Scand..

[28] Bäuerle A., Teufel M., Musche V., Weismüller B., Kohler H., Hetkamp M., Dörrie N., Schweda A., Skoda E.-M. (2020). Increased generalized anxiety, depression and distress during the COVID-19 pandemic: A cross-sectional study in Germany. J. Public Health.

[29] Taylor S., Landry C.A., Paluszek M.M., Fergus T.A., McKay D., Asmundson G.J.G. (2020). COVID stress syndrome: Concept, structure, and correlates. Depress. Anxiety.

[30] Bai Y.M., Lin C.C., Lin C.Y., Chen J.Y., Chue C.M., Chou P. (2004). Survey of stress reactions among health care workers involved with the SARS outbreak. Psychiatr Serv..

[31] Schäfer S.K., Sopp M.R., Schanz C.G., Staginnus M., Göritz A.S., Michael T. (2020). Impact of COVID-19 on public mental health and the buffering effect of a sense of coherence. Psychother. Psychosom..

[32] Peng E.Y.-C., Lee M.-B., Tsai S.-T., Yang C.-C., Morisky D.E., Tsai L.-T., Weng Y.-L., Lyu S.-Y. (2010). Population-based Post-crisis Psychological Distress: An Example From the SARS Outbreak in Taiwan. J. Formos. Med. Assoc..

[33] Li W., Yang Y., Liu Z.H., Zhao Y.J., Zhang Q., Zhang L., Cheung T., Xiang Y.T. (2020). Progression of mental health services during the COVID-19 outbreak in China. Int. J. Biol. Sci..

[34] Xiang Y.T., Yang Y., Li W., Zhang L., Zhang Q., Cheung T., Ng C.H. (2020). Timely mental health care for the 2019 novel coronavirus outbreak is urgently needed. Lancet Psychiatry.

[35] (2020). Living with COVID-19. https://evidence.nihr.ac.uk/themedreview/living-with-covid19/.

[36] Irons R. (2020). Pandemic … or syndemic? Re-framing COVID-19 disease burden and ‘underlying health conditions’. Soc. Anthropol..

[37] Ratcliff C.G., Barrera T.L., Petersen N.J., Sansgiry S., Kauth M.R., Kunik M.E., Stanley M.A., Cully J.A. (2017). Recognition of anxiety, depression, and PTSD in patients with COPD and CHF: Who gets missed?. Gen. Hosp. Psychiatry.

[38] Whooley M.A., De Jonge P., Vittinghoff E., Otte C., Moos R., Carney R.M., Ali S., Dowray S., Na B., Feldman M.D. (2008). Depressive symptoms, health behaviors, and risk of cardiovascular events in patients with coronary heart disease. JAMA-J. Am. Med. Assoc..

[39] Sokal J., Messias E., Dickerson F.B., Kreyenbuhl J., Brown C.H., Goldberg R.W., Dixon L.B. (2004). Comorbidity of Medical Illnesses Among Adults With Serious Mental Illness Who Are Receiving Community Psychiatric Services. J. Nerv. Ment. Dis..

[40] Mason B.W., Lyons R.A. (2003). Acute psychological effects of suspected bioterrorism. J. Epidemiol. Community Health.

[41] Gale S.D., Berrett A.N., Erickson L.D., Brown B.L., Hedges D.W. (2017). Association between virus exposure and depression in US adults. Psychiatry Res..

[42] van den Heuvel L., Chishinga N., Kinyanda E., Weiss H., Patel V., Ayles H., Harvey J., Cloete K.J., Seedat S. (2013). Frequency and correlates of anxiety and mood disorders among TB- and HIV-infected Zambians. AIDS Care-Psychol. Socio-Med. Asp. AIDS/HIV..

[43] Kuan V., Denaxas S., Gonzalez-Izquierdo A., Direk K., Bhatti O., Husain S., Sutaria S., Hingorani M., Nitsch D., Parisinos C.A. (2019). A chronological map of 308 physical and mental health conditions from 4 million individuals in the English National Health Service. Lancet Digit Health.

[44] Owens P.L., Heslin K.C., Fingar K.R., Weiss A.J. (2006). Co-Occurrence of Physical Health Conditions and Mental Health and Substance Use Conditions among Adult Inpatient Stays, 2010 Versus 2014: Statistical Brief #240. Healthcare Cost and Utilization Project (HCUP) Statistical Briefs. https://www.hcup-us.ahrq.gov/reports/statbriefs/sb240-Co-occurring-Physical-Mental-Substance-Conditions-Hospital-Stays.jsp.

[45] Singer M. (2020). Deadly Companions: COVID-19 and Diabetes in Mexico. Med. Anthr..

[46] Mendenhall E., Kohrt B.A., Norris S.A., Ndetei D., Prabhakaran D. (2017). Non-communicable disease syndemics: Poverty, depression, and diabetes among low-income populations. Lancet.

[47] Van Dooren F.E.P., Nefs G., Schram M., Verhey F.R.J., Denollet J., Pouwer F. (2013). Depression and Risk of Mortality in People with Diabetes Mellitus: A Systematic Review and Meta-Analysis. PLoS ONE.

[48] Chan J.W.M., Ng C.K., Chan Y.H., Mok T.Y.W., Lee S., Chu S.Y.Y., Law W.L., Lee M.P., Li P.C.K. (2003). Short term outcome and risk factors for adverse clinical outcomes in adults with severe acute respiratory syndrome (SARS). Thorax.

[49] Gerayeli F.V., Milne S., Cheung C., Li X., Yang C.W.T., Tam A., Choi L.H., Bae A., Sin D.D. (2021). COPD and the risk of poor outcomes in COVID-19: A systematic review and meta-analysis. EClinicalMedicine.

[50] CDC (2020). People with Moderate to Severe Asthma. Cent. Dis. Control Prev..

[51] Zhao Q., Meng M., Kumar R., Wu Y., Huang J., Lian N., Deng Y., Lin S. (2020). The impact of COPD and smoking history on the severity of COVID-19: A systemic review and meta-analysis. J. Med. Virol..

[52] Bruce M.A., Griffith D.M., Thorpe R.J. (2015). Stress and the kidney. Adv. Chronic Kidney Dis..

[53] Griffith D.M., Holliday C.S., Enyia O.K., Ellison J.M., Jaeger E.C. (2021). Using Syndemics and Intersectionality to Explain the Disproportionate COVID-19 Mortality Among Black Men. Public Health Rep..

[54] Padgett D.A., Glaser R. (2003). How stress influences the immune response. Trends Immunol..

[55] Ejaz H., Alsrhani A., Zafar A., Javed H., Junaid K., Abdalla A.E., Abosalif K.O., Ahmed Z., Younas S. (2020). COVID-19 and comorbidities: Deleterious impact on infected patients. J. Infect. Public Health.

[56] Ma L.-Y., Chen W.-W., Gao R.-L., Liu L.-S., Zhu M.-L., Wang Y.-J., Wu Z.-S., Li H.-J., Gu D.-F., Yang Y.-J. (2020). China cardiovascular diseases report 2018: An updated summary. J. Geriatr. Cardiol..

[57] Yang J., Zheng Y.A., Gou X., Pu K., Chen Z., Guo Q., Ji R., Wang H., Wang Y., Zhou Y. (2020). Prevalence of comorbidities and its effects in patients infected with SARS-CoV-2: A systematic review and meta-analysis. Int. J. Infect. Dis..

[58] Hardenberg J.H.B., Luft F.C. (2020). COVID-19, ACE2 and the kidney. Acta Physiol..

[59] Liu Y., Sun W., Li J., Chen L., Wang Y., Zhang L., Yu L. Clinical features and progression of acute respiratory distress syndrome in coronavirus disease 2019. medRxiv.

[60] Waliszewska-Prosół M., Budrewicz S. (2021). The unusual course of a migraine attack during COVID-19 infection—Case studies of three patients. J. Infect. Public Health.

[61] Straburzyński M., Kuca-Warnawin E., Waliszewska-Prosół M. COVID-19-related headache and innate immune response—A narrative review. Neurol. Neurochir. Pol..

[62] Sampaio Rocha-Filho P.A., Voss L. (2020). Persistent Headache and Persistent Anosmia Associated With COVID-19. Headache.

[63] Lechien J.R., Chiesa-Estomba C.M., Place S., Van Laethem Y., Cabaraux P., Mat Q., Huet K., Plzak J., Horoi M., Hans S. (2020). Clinical and epidemiological characteristics of 1420 European patients with mild-to-moderate coronavirus disease 2019. J. Intern. Med..

[64] Nicola M., Alsafi Z., Sohrabi C., Kerwan A., Al-Jabir A., Iosifidis C., Agha M., Agha R. (2020). The socio-economic implications of the coronavirus pandemic (COVID-19): A review. Int. J. Surg..

[65] Czeisler M.É., Marynak K., Clarke K.E., Salah Z., Shakya I., Thierry J.M., Ali N., McMillan H., Wiley J.F., Weaver M.D. (2022). Delay or Avoidance of Medical Care Because of COVID-19–Related Concerns—United States, June 2020. MMWR Morb. Mortal. Wkly. Rep..

[66] World Health Organization (WHO) (2019). Noncommunicable Diseases: Mortality. Glob Heal Obs [Internet]. https://www.who.int/data/gho/data/themes/topics/sdg-target-3_4-noncommunicable-diseases-and-mental-health.

[67] Czeisler M.É., Lane R.I., Petrosky E., Wiley J.F., Christensen A., Njai R., Weaver M.D., Robbins R., Facer-Childs E.R., Barger L.K. (2020). Mental Health, Substance Use, and Suicidal Ideation During the COVID-19 Pandemic—United States, June 24–30, 2020. MMWR Morb. Mortal. Wkly. Rep..

[68] Stokes E.K., Zambrano L.D., Anderson K.N., Marder E.P., Raz K.M., Felix S.E.B., Tie Y., Fullerton K.E. (2020). Coronavirus Disease 2019 Case Surveillance—United States, January 22–May 30, 2020. Morb. Mortal. Wkly. Rep..

[69] Williamson E.J., Walker A.J., Bhaskaran K., Bacon S., Bates C., Morton C.E., Curtis H.J., Mehrkar A., Evans D., Inglesby P. (2020). Factors associated with COVID-19-related death using OpenSAFELY. Nature.

[70] Gupta A., Madhavan M.V., Sehgal K., Nair N., Mahajan S., Sehrawat T.S., Bikdeli B., Ahluwalia N., Ausiello J.C., Wan E.Y. (2020). Extrapulmonary manifestations of COVID-19. Nat. Med..

[71] Bambra C., Riordan R., Ford J., Matthews F. (2020). The COVID-19 pandemic and health inequalities. J. Epidemiol. Commun. Health.

[72] Yadav U.N., Rayamajhee B., Mistry S.K., Parsekar S., Mishra S.K. (2020). A Syndemic Perspective on the Management of Non-communicable Diseases Amid the COVID-19 Pandemic in Low- and Middle-Income Countries. Front. Public Health.

[73] Sheldon T.A., Wright J. (2020). Twin epidemics of COVID-19 and non-communicable disease. BMJ.

[74] Pal R., Bhadada S.K. (2020). COVID-19 and non-communicable diseases. Postgrad. Med. J..

[75] Palmer K., Monaco A., Kivipelto M., Onder G., Maggi S., Michel J.-P., Prieto R., Sykara G., Donde S. (2020). The potential long-term impact of the COVID-19 outbreak on patients with non-communicable diseases in Europe: Consequences for healthy ageing. Aging Clin. Exp. Res..

[76] Hernández-Galdamez D.R., González-Block M.Á., Romo-Dueñas D.K., Lima-Morales R., Hernández-Vicente I.A., Lumbreras-Guzmán M., Mendez-Hernandez P. (2020). Increased Risk of Hospitalization and Death in Patients with COVID-19 and Pre-existing Noncommunicable Diseases and Modifiable Risk Factors in Mexico. Arch. Med. Res..

[77] Vegivinti C.T.R., Evanson K.W., Lyons H., Akosman I., Barrett A., Hardy N., Kane B., Keesari P.R., Pulakurthi Y.S., Sheffels E. (2022). Efficacy of antiviral therapies for COVID-19: A systematic review of randomized controlled trials. BMC Infect. Dis..

[78] Hua W., Xiaofeng L., Zhenqiang B., Jun R., Ban W., Liming L. (2020). The epidemiological characteristics of an outbreak of 2019 novel coronavirus diseases (COVID-19) in China. Zhonghua Liu Xing Bing Xue Za Zhi.

[79] Zaim S., Chong J.H., Sankaranarayanan V., Harky A. (2020). COVID-19 and Multiorgan Response. Curr. Probl. Cardiol..

[80] Wu Z., McGoogan J.M. (2020). Characteristics of and Important Lessons From the Coronavirus Disease 2019 (COVID-19) Outbreak in China: Summary of a Report of 72 314 Cases From the Chinese Center for Disease Control and Prevention. JAMA.

[81] Livingston E., Bucher K. (2020). Coronavirus Disease 2019 (COVID-19) in Italy. JAMA.

[82] Zhou P., Yang X.L., Wang X.G., Hu B., Zhang L., Zhang W., Si H.R., Zhu Y., Li B., Huang C.L. (2020). A pneumonia outbreak associated with a new coronavirus of probable bat origin. Nature.

[83] Arentz M., Yim E., Klaff L., Lokhandwala S., Riedo F.X., Chong M., Lee M. (2020). Characteristics and Outcomes of 21 Critically Ill Patients With COVID-19 in Washington State. JAMA.

[84] COVID Data Tracker Weekly Review|CDC. https://www.cdc.gov/coronavirus/2019-ncov/covid-data/covidview/index.html.

[85] Rosenthal N., Cao Z., Gundrum J., Sianis J., Safo S. (2020). Risk Factors Associated With In-Hospital Mortality in a US National Sample of Patients With COVID-19. JAMA Netw Open.

[86] People with Certain Medical Conditions|CDC. https://www.cdc.gov/coronavirus/2019-ncov/need-extra-precautions/people-with-medical-conditions.html.

[87] Excess Deaths Associated with COVID-19. https://www.cdc.gov/nchs/nvss/vsrr/covid19/excess_deaths.htm.

[88] 14.9 Million Excess Deaths Associated with the COVID-19 Pandemic in 2020 and 2021. https://www.who.int/news/item/05-05-2022-14.9-million-excess-deaths-were-associated-with-the-covid-19-pandemic-in-2020-and-2021.

[89] Islam N., Shkolnikov V.M., Acosta R.J., Klimkin I., Kawachi I., Irizarry R.A., Alicandro G., Khunti K., Yates T., Jdanov D.A. (2021). Excess deaths associated with COVID-19 pandemic in 2020: Age and sex disaggregated time series analysis in 29 high income countries. BMJ.

[90] Hedegaard H., Bastian B.A., Trinidad J.P., Spencer M., Warner M. (2018). Drugs most frequently involved in drug overdose deaths: United states, 2011–2016. Natl. Vital. Stat. Rep..

[91] Koob G.F., Powell P., White A. (2020). Addiction as a Coping Response: Hyperkatifeia, Deaths of Despair, and COVID-19. Am. J. Psychiatry.

[92] The Unrecognized Tragedy of Deaths of Despair-STAT. https://www.statnews.com/2021/12/29/deaths-of-despair-unrecognized-tragedy-working-class-immiseration/.

[93] Togun T., Kampmann B., Stoker N.G., Lipman M. (2020). Anticipating the impact of the COVID-19 pandemic on TB patients and TB control programmes. Ann. Clin. Microbiol. Antimicrob..

[94] Risk for COVID-19 Infection, Hospitalization, and Death by Race/Ethnicity|CDC. https://www.cdc.gov/coronavirus/2019-ncov/covid-data/investigations-discovery/hospitalization-death-by-race-ethnicity.html.

[95] (2020). Health Equity Considerations and Racial and Ethnic Minority Groups. https://stacks.cdc.gov/view/cdc/91049.

[96] Millett G.A., Jones A.T., Benkeser D., Baral S., Mercer L., Beyrer C., Honermann B., Lankiewicz E., Mena L., Crowley J.S. (2020). Assessing differential impacts of COVID-19 on black communities. Ann. Epidemiol..

[97] Cordes J., Castro M.C. (2020). Spatial analysis of COVID-19 clusters and contextual factors in New York City. Spat. Spatiotemporal. Epidemiol..

[98] Al Wahaibi A., Al-Maani A., Alyaquobi F., Al Harthy K., Al-Jardani A., Al Rawahi B., Al-Abri S. (2021). Effects of COVID-19 on mortality: A 5-year population-based study in Oman. Int. J. Infect. Dis..

[99] Singer M. (2010). Pathogen-pathogen interaction. Virulence.

[100] Shiau S., Krause K.D., Valera P., Swaminathan S., Halkitis P.N. (2020). The Burden of COVID-19 in People Living with HIV: A Syndemic Perspective. AIDS Behav..

[101] Mendenhall E. (2017). Syndemics: A new path for global health research. Lancet.

[102] Singer M., Bulled N., Ostrach B. (2020). Whither syndemics?: Trends in syndemics research, a review 2015–2019. Glob. Public Health.

[103] Singer M. (2000). A dose of drugs, a touch of violence, a case of AIDS: Conceptualizing the SAVA syndemic. Free Inq. Creat. Sociol..

[104] Hart L., Horton R. (2017). Syndemics: Committing to a healthier future. Lancet.

[105] Himmelgreen D.A., Romero-Daza N., Turkon D., Watson S., Okello-Uma I., Sellen D. (2009). Addressing the HIV/AIDS-food insecurity syndemic in sub-Saharan Africa. Afr. J. AIDS Res..

[106] Kwan C.K., Ernst J.D. (2011). HIV and Tuberculosis: A Deadly Human Syndemic. Clin. Microbiol. Rev..

[107] Pirrone I., Dieleman M., Reis R., Pell C. (2021). Syndemic contexts: Findings from a review of research on non-communicable diseases and interviews with experts. Glob. Health Action..

[108] Tsai A.C., Mendenhall E., Trostle J.A., Kawachi I. (2017). Co-occurring epidemics, syndemics, and population health. Lancet.

[109] Mendenhall E. (2020). The COVID-19 syndemic is not global: Context matters. Lancet.

[110] Mendenhall E. (2016). Beyond Comorbidity: A Critical Perspective of Syndemic Depression and Diabetes in Cross-cultural Contexts. Med. Anthropol. Q..

[111] Abu-Raddad L.J., Patnaik P., Kublin J.G. (2006). Dual infection with HIV and malaria fuels the spread of both diseases in Sub-Saharan Africa. Science.

[112] Gravlee C.C. (2020). Systemic racism, chronic health inequities, and COVID-19: A syndemic in the making?. Am. J. Hum. Biol..

[113] Horton R. (2020). Offline: COVID-19 is not a pandemic. Lancet.

[114] Pirtle W.N.L. (2020). Racial Capitalism: A Fundamental Cause of Novel Coronavirus (COVID-19) Pandemic Inequities in the United States. Health Educ. Behav..

[115] Mahase E. (2020). Coronavirus: COVID-19 has killed more people than SARS and MERS combined, despite lower case fatality rate. BMJ.

[116] Padmanabhan S., Padmanabhan S. (2021). The Coronavirus Disease 2019 (COVID-19) Pan-Syndemic—Will We Ever Learn?. Clin. Infect. Dis..

[117] Kang J.Y., Michels A., Lyu F., Wang S., Agbodo N., Freeman V.L., Wang S. (2020). Rapidly measuring spatial accessibility of COVID-19 healthcare resources: A case study of Illinois, USA. Int. J. Health Geogr..

[118] Weissman G.E., Crane-Droesch A., Chivers C., Luong T., Hanish A., Levy M.Z., Lubken J., Becker M., Draugelis M.E., Anesi G.L. (2020). Locally Informed Simulation to Predict Hospital Capacity Needs During the COVID-19 Pandemic. Ann. Intern. Med..

[119] Wollenstein-Betech S., Silva A.A.B., Fleck J.L., Cassandras C.G., Paschalidis I.C. (2020). Physiological and socioeconomic characteristics predict COVID-19 mortality and resource utilization in Brazil. PLoS ONE.

[120] How COVID, Inequality and Politics Make a Vicious Syndemic-Scientific American. https://www.scientificamerican.com/article/how-covid-inequality-and-politics-make-a-vicious-syndemic1/.

[121] Rálaigh C. (2021). What’s in a name? Applying the syndemic perspective to COVID-19 in Ireland. Ir. J. Sociol..

[122] An Echo Pandemic of Mental Health Issues? Not If We Can Help It.-CMHA National. https://cmha.ca/an-echo-pandemic-of-mental-health-issues-not-if-we-can-help-it/.

[123] Canadian Mental Health Association (2020). Policy Brief COVID-19 and Mental Health: Heading off an Echo Pandemic Introduction: The Impact of COVID-19 on Mental Health. https://cmha.ca/wp-content/uploads/2020/06/EN_COVID-19-Policy-Brief.pdf.

